# Multiple myeloma with t(11;14): impact of novel agents on outcome

**DOI:** 10.1038/s41408-023-00807-9

**Published:** 2023-03-20

**Authors:** Borja Puertas, Verónica González-Calle, Eduardo Sobejano-Fuertes, Fernando Escalante, Beatriz Rey-Bua, Irene Padilla, Ramón García-Sanz, Noemi Puig, Norma C. Gutiérrez, María-Victoria Mateos

**Affiliations:** 1 University Hospital of Salamanca/IBSAL/Cancer Research Center-IBMCC (USAL-CSIC), CIBERONC, Salamanca, Spain; 2 Department of Hematology, University Hospital Dr. José Molina Orosa, Lanzarote, Canary Islands, Palmas, Spain; 3 Department of Hematology, University Hospital of León, León, Spain

**Keywords:** Myeloma, Cancer therapy

## Abstract

Multiple myeloma (MM) patients with *t*(11;14) present unique biological features and their prognosis is not well established. We report a retrospective study of 591 MM patients, 17.3% of whom had *t*(11;14). It was designed to determine the prognostic impact of this abnormality and the effect of novel agents on the response and outcomes. Three groups were established based on their cytogenetics: (1) *t*(11;14); (2) high-risk chromosomal abnormalities; and (3) standard risk (SR). After 80.1 months (1.2–273.8 months) of follow-up, no differences were observed in overall survival (OS) between the *t*(11;14) and SR groups (75.8 vs. 87.2 months; *P* = 0.438). Treatment of MM *t*(11;14) with novel agents did not improve their overall response rate (ORR) or complete response (CR) compared with those who received conventional therapy (ORR: 87.2 vs. 79.5%, *P* = 0.336; CR: 23.4 vs. 12.8%, *P* = 0.215). This effect translated into a similar PFS (39.6 vs. 30.0 months; *P* = 0.450) and OS (107.6 vs. 75.7 months; *P* = 0.175). In summary, MM *t*(11;14) patients did not benefit from the introduction of novel agents as much as SR patients did, indicating that other therapies are needed to improve their outcomes.

## Introduction

Multiple myeloma (MM) is a neoplasm characterized by clonal plasma cell proliferation, with heterogeneous outcomes and treatment responses [1]. Chromosomal abnormalities detected by fluorescence in situ hybridization (FISH), especially *t*(4;14), *t*(14;16), and del17p, have been recognized as one of the most important prognostic factors of MM patients since the early 2000s [2–4]. Translocations involving chromosome 14, which includes the immunoglobulin heavy chain gene (*IGH*), are detected in ~60% of patients with newly diagnosed multiple myeloma (NDMM) [4]. The most common translocation in NDMM is *t*(11;14) (q13;q32), which is detected in 15–20% of myeloma patients [5–10]. This chromosomal abnormality is more frequent in AL amyloidosis and primary plasma cell leukemia (≈50% of patients of both entities) [11, 12]. Several authors have postulated that *t*(11;14) defines a subset of MM patients with unique biological characteristics. Lymphoplasmacytic morphology, increased numbers of circulating plasma cells, expression of CD20 in the tumor plasma cells, IgG lambda and Bence-Jones isotypes, oligosecretory (≤1 g/dL of M-component), and non-secretory disease are associated with MM *t*(11;14) [5, 13–16]. In addition, clonal plasma cells in this subgroup of MM patients exhibit a higher level of expression of the antiapoptotic protein BCL-2, and a lower level of expression of proapoptotic protein MCL-1/BCL-XL, making them susceptible to BCL-2 inhibition [17].

The prognostic impact of *t*(11;14) in NDMM patients remains unclear. The results of several studies carried out before and after the novel-agent era show that the prognosis associated with the presence of this translocation is evolving. In the pre-novel-agent era, many authors agreed that patients with MM and t(11;14) had a favorable outcome [5, 6, 8, 10, 18]. However, in the novel-agent era, although treatments such as proteasome inhibitors (PIs) and immunomodulators (IMIDs) have improved the survival of most patients with MM, it is not clear whether this benefit also accrues to patients with *t*(11;14) [15, 19–34].

In this scenario, this retrospective study was designed to estimate the prevalence of NDMM with *t*(11;14), to confirm that patients harboring this translocation are a subset with unique biological and clinical characteristics, and to investigate whether chemotherapy and novel agents might affect the outcome of MM with *t*(11;14).

## Methods

A retrospective observational study was conducted, enrolling patients correlatively diagnosed with MM at the University Hospital of Salamanca and the University Hospital of Leon between 1998 and 2018. The sample was restricted to the 591 patients who had been studied to detect the presence of the *t*(11;14) translocation. Between 1998 and 2005, FISH in non-separated clonal plasma cells was performed in patients with >10% bone marrow clonal plasma cell infiltration, as revealed by flow cytometry (76 patients, 12.9%) [18]. Since 2005, FISH studies in separated clonal plasma cells were carried out in 515 patients (87.1%), as previously reported [35]. Patients were analyzed for illegitimate rearrangements of the *IGH* gene, *t*(11;14), *t*(4;14), and *t*(14;16), deletion 17p (del17p), 1q gain, and 1p deletion. A threshold of 10% was used as the cut‐off for translocations and 20% for numerical aberrations. The cut-off date for follow-up was 31 January 2022. The ethical committee of the University Hospital of Salamanca approved the study, which was conducted in accordance with the 1964 Declaration of Helsinki.

Patients were classified into three cytogenetic groups: (1) the *t*(11;14) group, which includes patients with this chromosomal abnormality without del17p; (2) the high-risk chromosomal abnormalities (HRCA) group, which includes patients with *t*(4;14), *t*(14;16), and del17p, and includes patients with del17p plus *t*(11;14); and (3) the standard risk (SR) group, which includes patients without any of the aforementioned cytogenetic abnormalities. Patients solely with chromosome 1 alterations are also included in this group.

The treatment strategies adopted were divided into those involving novel agents, which included PI, IMIDs, and anti-CD38 monoclonal antibodies, and those based on conventional therapies, such as chemotherapy (melphalan or cyclophosphamide with dexamethasone/prednisone) and polychemotherapy (e.g., the combination of vincristine, carmustine, melphalan, cyclophosphamide, and prednisone; VBCMP). Two categories of novel-agent combinations were established based on the number of drugs they contained. One novel-agent combination covers those containing a single novel agent with other drugs. They are generally doublets, like bortezomib plus dexamethasone (VD) or lenalidomide plus dexamethasone (RD), but can also be triplets, like bortezomib plus cyclophosphamide and dexamethasone (VCD). A group of combinations with at least two novel agents was also considered. Patients who received two or more novel agents (usually as triplets), like bortezomib plus lenalidomide and dexamethasone (VRD), or patients treated with daratumumab, bortezomib, melphalan, and dexamethasone (D-VMP) were included in this group.

Responses were assessed in accordance with the 2014 International Myeloma Working Group (IMWG) criteria [36]. Overall rate response (ORR) was defined as the percentage of patients who achieved partial response (PR) or better. Complete response and stringent complete response were pooled to create a single complete response (CR) category. Both responses were evaluated after induction to avoid the effect of a high dose of melphalan in transplant-eligible patients.

OS was defined as the time from diagnosis until the date of death or last follow-up. PFS was defined as the time from diagnosis until clinical progression or death, whichever occurred first.

A descriptive analysis was carried out to summarize the cohort of MM patients included in the study. The statistically significant differences in the qualitative and quantitative variables between the *t*(11;14) and other cytogenetic groups were estimated using the chi-square and Student *t*-tests, respectively. The distributions of OS and PFS were estimated by the Kaplan–Meier method. The log-rank test was used to determine statistically significant differences between the survival of different subgroups of patients. Univariate and multivariate Cox regressions were carried out. Values of *P* < 0.05 were considered significant for all statistical tests. Analyses were performed with IBM SPSS Statistics, version 26.

## Results

**Fig. 1:**
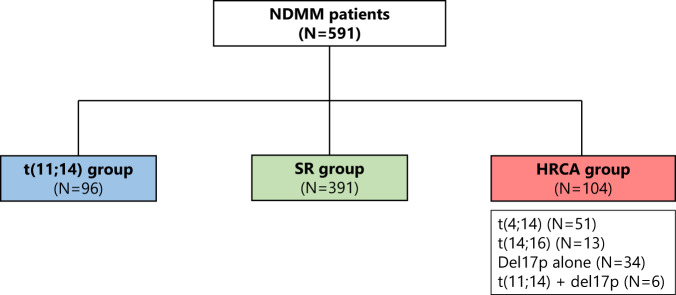
Cytogenetic group. NDMM newly diagnosed multiple myeloma, SR standard risk, HRCA high-risk chromosomal abnormalities.

One hundred and two (17.3%) of the 591 patients in the cohort harbored the *t*(11;14) translocation. Ninety-six (16.2%) belonged to the *t*(11;14) group, 391 (66.2%) to the SR group, and 104 (17.6%) to the HRCA group. Within the HRCA group, 51 (8.6%) had *t*(4;14), 13 (2.2%) had *t*(14;16), and 34 (5.8%) had solely del17p. Six patients with co-occurring del17p and *t*(11;14) were also assigned to the HRCA group (Fig. 1).

The overall cohort had a median age at diagnosis of 67 years (28–96 years), and 344 (58.2%) of the cohort were men. The clinical and biological characteristics of the MM patients in the study are summarized in Table 1. As an induction treatment, 357 patients (63.6%) were treated with novel agents and 204 (36.4%) received conventional therapy (Table 2). Four patients did not receive any treatment (therapeutic abstention due to performance status), and the treatment of 26 patients was unknown. Overall, 269 (45.6%) patients underwent ASCT.

**Table 1:** Clinical and biological characteristics of newly diagnosed multiple myeloma patients.

	MM *t*(11;14) (*N* = 96)	MM SR (*N* = 391)	*P* value	MM *t*(11;14) (*N* = 96)	MM HRCA (*N* = 104)	*P* value
Age at diagnosis (median, range)	68 (32–89)	67 (29–96)	0.762	68 (32–89)	65 (28–85)	0.083
Sex, male (no. %)	51 (53.1)	229 (58.7)	0.304	51 (53.1)	64 (61.5)	0.229
Ig subtype (no. %)						
IgG	57 (60.0)	227 (59.6)	0.940	57 (60.0)	52 (50.0)	0.157
IgA	**12 (12.6)**	**94 (24.7)**	**0.012**	**12 (12.6)**	**32 (30.8)**	**0.002**
IgM	**1 (1.1)**	**0 (0.0)**	**0.045**	**1 (1.1)**	1 (1,00)	0.949
IgD	1 (1.1)	1 (0.3)	0.287	1 (1.1)	0 (0.0)	0.294
Non-secretory	**10 (10.5)**	**6 (1.6)**	**0.000**	**10 (10.5)**	**3 (2.90)**	**0.028**
Light chains only	14 (14.7)	53 (13.9)	0.836	14 (14.7)	16 (15.4)	0.894
Light-chain subtype (no. %)						
Kappa	**49 (51.6)**	**239 (63.6)**	**0.032**	49 (51.6)	50 (48.5)	0.670
Lambda	36 (37.9)	131 (34.8)	0.578	36 (37.9)	50 (48.5)	0.131
≥60% PC in BM (no. %)	**26 (31.3)**	**49 (14.2)**	**0.000**	26 (31.3)	21 (21.9)	0.152
Paraprotein ≤1 g/dL (no. %)	17 (23.6)	42 (15.2)	0.088	17 (23.6)	15 (19.7)	0.567
Bence-Jones proteinuria (g/24 h) (mean, SD)	1.73 (±3.74)	1.38 (±2.54)	0.436	1.73 (±3.74)	2.53 (±4.05)	0.303
Albumin (g/dL) (mean, SD)	3.62 (±0.70)	3.62 (±0.70)	0.977	3.62 (±0.70)	3.43 (±0.71)	0.076
β2-microglobulin (g/dL) (mean, SD)	4.50 (±3.09)	5.22 (±4.17)	0.077	**4.50 (±3.09)**	**6.44 (±5.03)**	**0.002**
Corrected calcium (mg/dL) (mean, SD)	**9.97 (±1.36)**	**9.62 (±1.37)**	**0.035**	9.97 (±1.36)	10.0 (±1.82)	0.845
Hemoglobin (g/L) (mean, standard deviation)	11.1 (±1.88)	11.1 (±2.22)	0.840	**11.1 (±1.88)**	**10.6 (±1.87)**	**0.046**
Creatinine (mg/dL) (mean, SD)	1.35 (±1.18)	1.46 (±1.50)	0.451	1.35 (±1.18)	1.66 (±1.77)	0.155
Elevated LDH (no. %)	31 (46.3)	102 (34.8)	0.080	31 (46.3)	36 (50.0)	0.660
Lytic bone lesions (no. %)	52 (60.5)	209 (63.0)	0.671	52 (60.5)	58 (59.2)	0.860
Plasmacytomas (no. %)	**10 (17.2)**	**96 (38.6)**	**0.002**	**10 (17.2)**	**27 (37.5)**	**0.011**
ECOG PS 0–1 (no. %)	59 (72.8)	212 (72.1)	0.897	59 (72.8)	58 (69.0)	0.592
ISS (no. %)						
I	30 (33.0)	128 (35.8)	0.619	30 (33.0)	27 (26.5)	0.323
II-III	61 (67.0)	230 (64.2)	0.619	61 (67.0)	75 (73.5)	0.323

*MM* multiple myeloma, *Ig* immunoglobulin, *PC* plasma cell, *BM* bone marrow, *LDH* lactate dehydrogenase, *ECOG* Eastern Cooperative Oncology Group, *PS* performance status, *ISS* International staging system, *SR* standard risk, *HRCA* high-risk chromosomal abnormalities, *SD* standard derivation.

Bold values indicates statistical significant *P* values.

**Table 2:** First-line treatment of newly diagnosed multiple myeloma patients.

	MM *t*(11;14) (*N* = 96)	MM SR (*N* = 391)	MM HRCA (*N* = 104)	*P* value
Number of treatment lines (median, range)	2 (0–8)	2 (0–14)	2 (1–8)	No statistically significant differences were observed
**Conventional treatment induction scheme**				
• Conventional treatment in the first line	44.4% (40 of 90)	34.4% (128 of 372)	36.4% (36 of 99)	
• Conventional chemotherapy (CyP, MP)	22.2% (20 of 90)	14.4% (54 of 372)	13.2% (13 of 99)	
• Polychemotherapy (VBCMP, VBAD, VAD)	22.2% (20 of 90)	20.0% (74 of 372)	23.2% (23 of 99)	
**Novel agents – based induction schemes**				
• Novel agents in the first line	55.6% (50 of 90)	65.6% (244 of 372)	63.6% (63 of 99)	
• 1 Novel agent in the first line	42.2% (38 of 90)	43.8% (163 of 372)	39.4% (39 of 99	
• ≥2 novel agents in the first line	13.4% (12 of 90)	21.8% (81 of 372)	24.2% (24 of 99)	
• PI-based regimens (VD, VMP, VCD, PAD…)	38.9% (35 of 90)	38.7% (144 of 372)	35.4% (35 of 99)	
• IMID-based regimens (TD, TCD, TAD, Rd..)	3.3% (3 of 90)	5.6% (21 of 372)	4.0% (4 of 99)	
• PI plus IMID (VTD, VRD…)	11.1% (10 of 90)	18.5% (69 of 372)	19.2% (19 of 99)	
• Anti-CD38-based regimens	2.2% (2 of 90)	2.7% (10 of 372)	5.1% (5 of 99)	
ASCT	43.2% (41 of 95)	44.8% (175 of 391)	51.0% (53 of 104)	

*MM* multiple myeloma, *HRCA* high-risk chromosomal abnormalities, *PI* proteasome inhibitor, *IMID* immunomodulator, *CyP* cyclophosphamide and prednisone, *MP* melphalan prednisone, *VBCMP* vincristine, carmustine, cyclophosphamide, melphalan, and prednisone, *VBAD* vincristine, bleomycin, adriamycin, and dexamethasone, *VAD* vincristine, adriamycin, and dexamethasone, *VD* bortezomib and dexamethasone, *VMP* bortezomib, melphalan, and dexamethasone, *VCD* bortezomib, cyclophosphamide, and dexamethasone, *PAD* bortezomib, adriamycin, and dexamethasone *TD* thalidomid and dexamethasone, *TCD* thalidomide, cyclophosphamide, and dexamethasone, *TAD* thalidomide, adriamycin, and dexamethasone, *Rd* lenalidomide and dexamethasone, *VTD* bortezomib, thalidomide, and dexamethasone, *VRD* bortezomib, lenalidomide, and dexamethasone, *ASCT* autologous stem cell transplantation.

### Clinical and biological features of patients with *t*(11;14)

Compared with the SR group, fewer patients in the *t*(11;14) had the IgA subtype (12.6 vs. 24.7%; *P* = 0.012) or the light-chain kappa (51.6 vs. 63.6%; *P* = 0.032), while a significantly greater proportion of patients had the non-secretory disease (10.5 vs. 1.6%; *P* = 0.000) and, although not significantly, more had oligosecretory disease (23.6 vs. 15.2%; *P* = 0.088). In addition, the presence of at least 60% plasma cells in bone marrow (31.3 vs. 14.2%; *P* = 0.000), higher serum calcium levels (9.97 mg/dL ± 1.36 vs. 9.62 mg/dL ± 1.37; *P* = 0.035), and a lower incidence of plasmacytomas (including paraskeletal and extramedullary types) (17.2 vs. 38.6%; *P* = 0.002) were observed in the *t*(11;14) group.

When comparing *t*(11;14) with the HRCA group, there were also fewer IgA patients in the *t*(11;14) group (12.6 vs. 30.8%; *P* = 0.002), and more non-secretory patients (10.5 vs. 2.9%; *P* = 0.028). Furthermore, the incidence of plasmacytomas (17.2 vs. 37.5%; *P* = 0.011), and the β_2_ microglobulin level were lower at diagnosis in the *t*(11;14) than in the HRCA group (4.49 mg/L ± 3.08 vs. 6.44 mg/L ± 5.03; *P* = 0.002). In contrast, patients with *t*(11;14) had higher levels of hemoglobin compared with the HRCA group (11.1 g/dL ± 1.88 vs. 10.6 g/dL ± 1.87; *P* = 0.046).

No other statistically significant differences in baseline characteristics were observed when comparing the *t*(11;14), SR, and HRCA cytogenetic groups (Table 1).

### Progression-free survival and overall survival of MM patients with *t*(11;14)

After a median follow-up of 80.1 months (range, 1.2–273.8 months), 239 (41.1%) patients were still alive at the last contact. The median PFS and OS of the whole cohort was 28.7 months (95% confidence interval (CI), 25.4–31.9 months) and 72.5 months (95% CI, 59.8–85.1 months), whereas those of patients with *t*(11;14) were 35.3 months (95% CI, 23.8–46.7 months) and 75.8 months (95% CI, 45.2–106.3 months), respectively. The univariate associations with the outcome of age at diagnosis, baseline characteristics (those in Table 1), achieving ORR and CR, receiving new agents in the first line, and undergoing ASCT were calculated. The significant variables were then considered in a multivariate model. Not achieving CR after induction and β_2_ microglobulin ≥5.0 mg/dL were identified as poor prognostic factors for PFS, and not undergoing ASCT was a poor prognostic factor for PFS and OS in the *t*(11;14) group. Age ≥65 years at diagnosis and not achieving ORR after induction were identified as independent poor prognostic factors for PFS and OS (Supplementary Table 1).

**Fig. 2:**
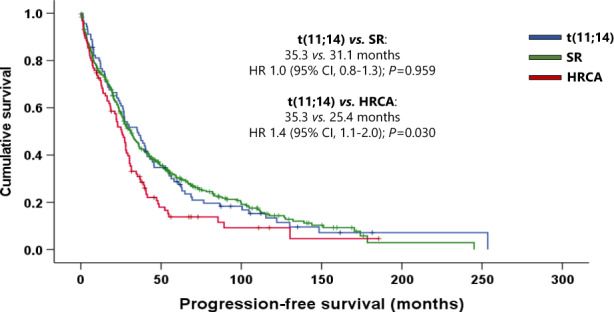
Progression-free survival by a cytogenetic group. SR standard risk, HRCA high-risk chromosomal abnormalities, HR hazard ratio, CI confidence interval.

**Fig. 3:**
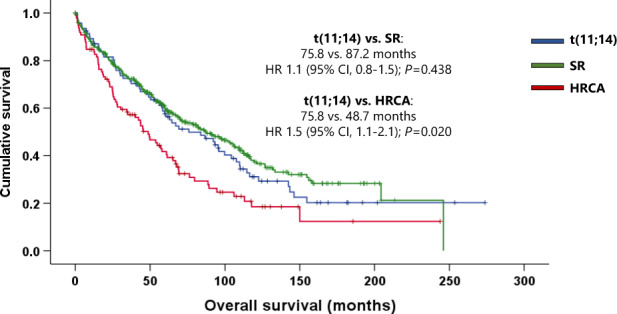
Overall survival by a cytogenetic group. SR standard risk, HRCA high-risk chromosomal abnormalities, HR hazard ratio, CI confidence interval.

Comparison of *t*(11;14) patients with the SR group revealed no statistically significant differences in PFS (35.3 vs. 31.1 months; hazard ratio (HR) 1.0 [95% CI, 0.8–1.3]; *P* = 0.959), or OS (75.8 vs. 87.2 months; HR 1.1 [95% CI, 0.8–1.5]; *P* = 0.438). However, the median PFS and OS were significantly longer in the *t*(11;14) group than in the HRCA group: 35.3 vs. 25.4 months (HR 1.4 [95% CI, 1.1–2.0); *P* = 0.030) and 75.8 vs. 48.7 months (HR 1.5 [95% CI, 1.1–2.1]; *P* = 0.020), respectively (Figs. 2, 3).

### Response to novel drugs and their impact on survival in MM with t(11;14)

The ORR of the entire cohort and of patients with *t*(11;14) were 84.5 and 83.7%, while 27.0 and 18.6% of patients achieved CR, respectively.

Since no differences in PFS or OS were identified between the *t*(11;14) and SR groups, we explored the potential effect of the treatments received as induction in both groups.

**Fig. 4:**
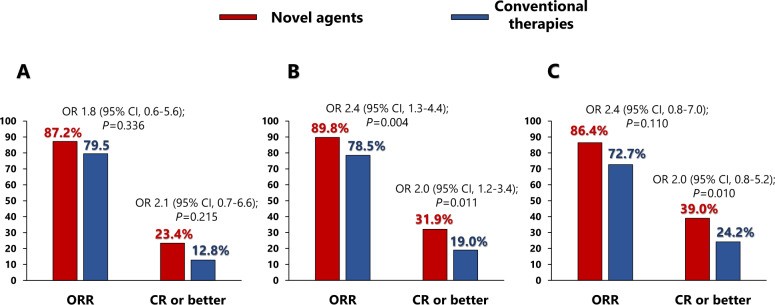
Response to treatment after induction divided by cytogenetic group. **A** Response to treatment in the t(11;14) group. **B** Response to treatment in the standard group. **C** Response to treatment in the high-risk chromosomal abnormalities group.

The use of novel agents in first line showed a non-significant trend towards a better response in the *t*(11;14) group. No significant differences were observed between those who received novel (87.2%) or conventional (79.5%) agents (odds ratio (OR) 1.8 [95% CI, 0.6–5.6]; *P* = 0.336). Neither did the CR rate differ significantly (23.4% vs. 12.8%; OR 2.1 [95% CI, 0.7–6.6]; *P* = 0.215). However, the SR group achieved a significantly better response with novel agents than with conventional therapies: ORR 89.8% vs. 78.5%, respectively (OR 2.4 [95% CI, 1.3–4.4]; *P* = 0.004). A higher percentage of those treated with novel agents achieved CR than did those receiving conventional therapies (31.9% vs. 19.0%; OR 2.0 [95% CI, 1.2–3.4]; *P* = 0.011). In the case of the HRCA group, despite a trend towards better responses in patients treated with novel agents, no significant differences were observed between those treated with new drugs and with conventional treatments in terms of ORR (86.4% vs. 72.7%; OR 2.4 [95% CI, 0.8–7.0]; *P* = 0.110) or CR (39.0% vs. 24.2%; OR 2.0 [95% CI, 0.8–5.2]; *P* = 0.155) (Fig. 4).

Patients across the entire cohort treated with novel agents in first line achieved significantly better PFS and OS than did patients receiving conventional therapies, 31.1 vs. 27.3 months (HR 1.3 [95% CI, 1.1–1.6]; *P* = 0.010) and 93.2 vs. 66.6 months (HR 1.4 [95% CI, 1.1–1.7]; *P* = 0.003), respectively. In contrast, although patients with *t*(11;14) treated with novel agents had longer PFS and OS than those receiving conventional therapies, neither of these differences was statistically significant (PFS: 39.6 vs. 30.0 months; HR 1.2 [95% CI, 0.8–1.9]; *P* = 0.450. OS: 107.6 vs. 75.7 months; HR 1.4 [95% CI, 0.8–2.4]; *P* = 0.175).

As the novel agents have gradually been incorporated into the treatment landscape, we decided to evaluate whether one compared with two or more novel agents in the first line produced different outcomes. Three hundred and fifty-seven of the 591 patients were treated with novel agents at the first line, 67.2% of whom received one novel-agent combination (usually as duplets), and 32.8% received at least two novel-agent combinations (usually as triplets) (Table 2). Across the entire cohort, patients who received at least two novel agents (*N* = 117) presented significantly better PFS (48.7 months) and OS (143.3 months) than did patients who received a single novel-agent combination (*N* = 240) (25.7 and 67.0 months, respectively) (HR 1.7 [95% CI, 1.3–2.3]; *P* = 0.000; HR 1.7 [95% CI, 1.2–2.5]; *P* = 0.003; respectively).

**Fig. 5:**
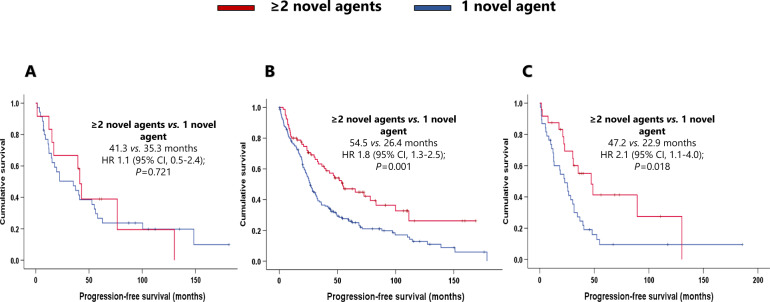
Effect of the combination of at least two novel agents in the first line in progression-free survival. **A** Effect of the combination of at least two novel agents in the first line in progression-free survival in the t(11;14) group. **B** Effect of the combination of at least two novel agents in the first line in progression-free survival in the standard risk group. **C** Effect of the combination of at least two novel agents in the first line in progression-free survival in the high-risk chromosomal abnormalities group.

**Fig. 6:**
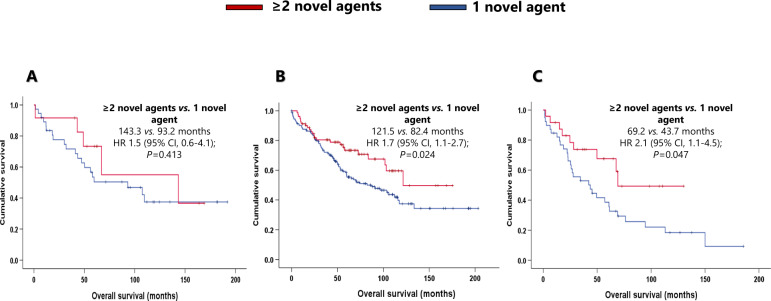
Effect of the combination of at least two novel agents in the first line in overall survival. **A** Effect of the combination of at least two novel agents in the first line in overall survival in the t(11;14) group. **B** Effect of the combination of at least two novel agents in the first line in overall survival in the standard risk group. **C** Effect of the combination of at least two novel agents in the first line in overall survival in the high-risk chromosomal abnormalities group.

In the *t*(11;14) group, patients treated with at least two novel agents (*N* = 12) had better survival, but not significantly longer PFS (41.3 months) or OS (143.3 months) than those who received a single novel agent (*N* = 38) in the induction (PFS: 35.3 months; HR 1.1 [95% CI, 0.5–2.4]; *P* = 0.721. OS, 93.2 months; HR 1.5 [95% CI, 0.6–4.1]; *P* = 0.413). The median PFS and OS of SR patients treated with at least two novel agents (*N* = 81) were 54.5 months and 121.5 months, respectively. These outcomes were significantly better than those of patients who received a single novel agent (*N* = 163) (median PFS: 26.4 months; HR 1.8 [95% CI, 1.3–2.5]; *P* = 0.001. Median OS: 82.4 months; HR 1.7 [95% CI, 1.1–2.7]; *P* = 0.024). Within the HRCA group, patients who received at least two novel agents (*N* = 24) had a longer PFS and OS than those treated with one novel agent (*N* = 39) (PFS: 47.2 vs. 22.9 months; HR 2.1 [95% CI, 1.1–4.0]; *P* = 0.018. OS: 69.2 vs. 43.7 months; HR 2.1 [95% CI, 1.1–4.5]; *P* = 0.047) (Figs. 5, 6).

The introduction of novel agents in the first line has not led to significantly better response and survival in patients with *t*(11;14) relative to the other cytogenetic groups, and their survival is no different from that of patients in the SR group. This called into question whether patients who harbored this translocation were more sensitive to chemotherapy. The effect of high doses of melphalan was analyzed in transplant-eligible patients by measuring the percentages of patients who had reached CR at day 100 after ASCT, and of patients who had a better post-transplant than pre-transplant response. Patients who achieved CR before transplantation were excluded because there were no instances of minimal residual disease response. There were no statistically significant differences in the percentages of CR at day 100 after ASCT of t(11;14) patients (28.1%) compared with the SR group (24.3%) (*P* = 0.663) or with the HRCA group (41.4%) (*P* = 0.277). Likewise, no differences were noted in the percentage of patients whose response improved after transplant: 50.0% of patients in the t(11;14) group, 46.8% of SR patients (*P* = 0.753), and 51.7% of HRCA patients (*P* = 0.893).

## Discussion

In this retrospective study of 591 NDMM patients diagnosed over two decades, we found a prevalence of *t*(11;14) (17.3%), consistent with other studies [5, 6, 8, 10, 18, 20, 22, 27, 30, 32]. We also noted that this group of patients represents a subgroup of MM with unique biological and clinical characteristics, with outcomes similar to those of the SR group but benefiting less from the introduction of novel agents in the first line in terms of response and PFS.

Several studies have shown that primary cytogenetic abnormalities redefine MM as a compendium of neoplasms with different biological characteristics, clinical features, and responses to treatment [37]. In the present study, we focused on one of these MM subtypes: the *t*(11;14). Some studies have attempted to analyze the prognostic significance of *t*(11;14) in terms of response and survival [5, 8, 24, 25, 32], but, to the best of our knowledge, this is the first to explore the value of *t*(11;14) as a predictor of response to new drugs or chemotherapy-containing regimens in the first line as well as the prognostic value in the era of novel agents, in a representative and unique series, because almost half of the patients received chemo-schemes and the others received novel agents.

This study confirmed that patients with MM and *t*(11;14) make up a singular subtype of myeloma, as first described by Fonseca et al. [5]. *t*(11;14) patients are characterized, in comparison to SR patients, by lower incidences of IgA, and of plasmacytomas, but a higher probability of having oligosecretory and non-secretory disease, higher PC bone marrow infiltration and levels of serum calcium. However, patients with *t*(11;14) had a less aggressive phenotype than those with HRCA, that is typified by fewer plasmacytomas, a lower β_2_ level, and a higher hemoglobin level. Other characteristic features of *t*(11;14), such as being diagnosed at a young age (≤50 years) [38], and the presence of Bence-Jones [7, 16], IgE (16), or IgD subtypes [20], were not disproportionally represented in our cohort. Lymphoplasmacytic morphology [13, 14, 20], the greater abundance of CD20 [14, 20], the lesser abundance of CD56 [20] in clonal PCs, and higher levels of circulating PCs [39] were not analyzed in the present study.

The prognostic significance of *t*(11;14) is controversial. Some studies compared groups with and without *t*(11;14). The latter group included high-risk patients, which could have favored the group with *t*(11;14) and could explain why it was considered to have a good prognosis, especially in the pre-novel-agent era [6–8, 10]. To avoid this, we considered three groups (*t*(11;14), high-risk, and all others considered to be standard risk) and analyzed the impact of chemo/novel-agent induction. In our series, when comparing these cytogenetics groups, patients of the *t*(11;14) group presented a similar PFS and OS to those of the SR group. However, MM *t*(11;14) showed better survival than the HRCA group. These results are supported by reports from several series [24, 31, 32].

Our results about the treatment response within the t(11;14) group are consistent with those previously published. Fonseca et al. [7] and Kumar et al. [28] obtained similar results of ORR with chemotherapy-containing induction in the t(11;14) population as we found in our cohort (73.6% vs. 76.0 and 80.0%, respectively). In our study, PR or better was achieved in almost 90% of patients with *t*(11;14) treated with novel agents, such as the ORR reported by Saini et al. [26] (93.7%), although lower ORR were reported by other groups, like those of Lakshman et al. [24] (73.6%), or Kumar et al. [28] (79.0%). In addition, Takamatsu et al. [15] evaluated the CR with novel agents in the *t*(11;14) subtype, obtaining a value of 38.0% that was slightly better than ours (23.4%). Unexpectedly, the introduction of new drugs did not improve the ORR or the percentage of CR in our series of *t*(11;14) patients. Conversely, an improvement was observed in the SR group. These findings are in line with the results of other authors who found that *t*(11;14) patients did not benefit as much from new treatments as did other myeloma subtypes [20, 24, 25, 33, 34].

The prognostic role of *t*(11;14) is evolving. In the pre-novel agent era, *t*(11;14) was considered a good prognostic factor, possibly because of its chemosensitivity, which was attributed to its lymphoplasmacytic morphology [6]. Furthermore, the resistance to melphalan is not mediated by BCL-2 overexpression [40]. Our study aimed to investigate the increased sensitivity to chemotherapy in *t*(11;14) by focusing on the “isolated” effect of melphalan in transplant-eligible patients, comparing responses immediately before transplant (after induction) with those achieved by day 100 after transplant, excluding patients who had achieved CR before transplantation since no minimal residual disease response was available for most subjects. The response of patients of the *t*(11;14) group did not improve after the transplant any more than it did in the other cytogenetic groups. These results are consistent with those of Moreau et al. [6] and Gao et al. [31]. Therefore, *t*(11;14) was not a predictive biomarker of response to chemotherapy in our cohort.

However, this abnormality was considered an intermediate prognosis translocation in the era of novel drugs [21, 22, 24–26]. Suboptimal response to novel-agent inductions in *t*(11;14) patients was one of the foremost reasons for this conclusion. Kaufman et al. [25] and Pirmohamed et al. [33] undertook retrospective studies of patients homogenously treated with VRD, both of which showed that *t*(11;14) patients had a worse response to induction and, consequently, worse PFS than SR patients. In addition, a subanalysis conducted within the Spanish GEM2005MENOS65 and GEM2012 trials of transplant-eligible patients revealed similar responses and outcomes for patients with *t*(11;14) and SR when they received conventional chemotherapy. However, the efficacy was lower for patients with *t*(11;14) when treated with novel schemes, such as VTD or VRD [34].

Gasparetto et al. recently published findings from the Connect MM registry [32], in which 24% of the cases were *t*(11;14) patients, and most were treated with novel agents in the first line. The median PFS and OS of the *t*(11;14) group were 34.3 and 83.2 months, respectively. These results are consistent with our series, but no statistically significant differences in PFS or OS between *t*(11;14) and SR patients were noted, suggesting that, in the era of IMID/PI treatments, *t*(11;14) has a neutral prognosis. Another study, conducted by the IMWG, and which included more than 800 *t*(11;14) patients, showed that those who received a PI plus IMID combination had better outcomes than those treated with PI or IMID alone, and that upfront ASCT resulted in survival close to 10 years [28].

In our series, patients from the *t*(11;14) group treated with novel agents showed a trend towards better response and survival. However, the introduction of new drugs has not led to a significant improvement in the outcomes of these patients, as observed in the SR and HRCA groups, especially those who received two or more novel agents. Although the OS of patients with *t*(11;14) treated with novel agents is numerically longer, the difference was not statistically significant. The sample size of MM with *t*(11;14) treated with at least two drugs (*N* = 12) and the new treatments in the relapse setting, such as anti-CD38 monoclonal antibodies, bispecific monoclonal antibodies, and T lymphocytes with chimeric antigen receptors, could have influenced the results. Therefore, this translocation could be considered a marker of non-response to new treatments such as those with PIs or IMIDs. We suggest that the prognostic significance of this translocation is neutral, as the survival of patients with *t*(11;14) was comparable to that of the SR group, even though the new agents did not seem to improve their outcomes.

To explain why PIs and IMIDs are less effective in *t*(11;14), we can consider the expectations arising from the endoplasmic reticulum stress theory [41]. This explains how compensatory pathways, such as the unfolded protein response, by activating the proteasome, are activated when PCs accumulate unfolded or misfolded proteins to eliminate them. However, clonal PCs that harbor *t*(11;14) have a lymphoplasmacytic morphology, with scant cytoplasm and less rough endoplasmic reticulum. For this reason, these PCs might be less likely to accumulate proteins and less able to activate the compensatory mechanism derived from endoplasmic reticulum stress, and thereby less susceptible to drugs that inhibit it directly (e.g., PIs [41]) or indirectly (e.g., IMIDs [42]).

The BCL-2 protein family is well known to play a critical role in the apoptosis of clonal PCs. Treatment with BCL-2 inhibitors, like venetoclax, is emerging as the first targeted therapy for MM, because it is more effective in cases of MM with a high level of expression of BCL-2 and a low level of MCL-1/BCL-XL expression, such as the *t*(11;14) profile [43–45]. No targeted therapy has yet been approved for the treatment of MM, because in the phase 3 BELLINI trial [46], relapsed/refractory MM patients randomized to the venetoclax arm exhibited higher mortality. Furthermore, the venetoclax group had a significantly better response and PFS in patients with *t*(11;14) and/or BCL-2 overexpression. Based on these promising results, the role of BCL-2 inhibitors in the *t*(11;14) group is being investigated in ongoing trials. Results are awaited for the phase 3 CANOVA study, which has recruited only relapsed/refractory MM patients with *t*(11;14) and randomized them to venetoclax-dexamethasone versus pomalidomide-dexamethasone [47]. Based on these results and those of other studies, it is not clear whether the current treatment recommended for NDMM is optimal for patients with *t*(11;14). However, anti-BCL-2 drugs are particularly effective in patients with *t*(11;14), so it will be worthwhile investigating anti-BCL-2 targeted therapy approaches in the upfront setting in MM.

The present study has several limitations, most notably its retrospective nature and the heterogeneity of the induction schemes. The potential for type 2 errors means that the results of subgroup analyses featuring small numbers of patients should be interpreted with caution, especially when comparing subgroups of patients who received at least two novel agents. However, as mentioned before, the study is of particular value because the inclusion of patients with MM diagnosed over 20 years makes this series unique.

In conclusion, MM with *t*(11;14) represents a subset of patients with unique clinical biological characteristics, with more bone marrow infiltration, less protein secretion, and fewer plasmacytomas at diagnosis. The survival of patients with *t*(11;14) was not worse than that of the SR group, but better than that of the HRCA group. The introduction of novel agents in the first line did not benefit the *t*(11;14) group as much as the other cytogenetic subgroups in terms of response, PFS, or OS, so this translocation may be considered a marker of suboptimal response to IMIDs/PIs. Further studies with new treatments such as venetoclax are necessary if the outcome of *t*(11;14) patients is to be improved.

## Supplementary information

Supplementary material

## Data Availability

The data that support the findings of this study are available from the corresponding author (M-MV), upon reasonable request.
