# Supplementary material for: Multiple myeloma with t(11;14): impact of novel agents on outcome
Source: Blood Cancer J. 2023 Mar 20;13(1):40. doi: 10.1038/s41408-023-00807-9 (PMC10025259; doi:10.1038/s41408-023-00807-9)
Supplement: Supplementary file 1 — Supplementary material [file 41408_2023_807_MOESM1_ESM.docx]

**Supplementary data legends**

**Supplementary table 1.** Univariate and multivariate analysis in the t(11;14) group

|  | **Progression-free survival** | | | |  | **Overall survival** | | | |
| --- | --- | --- | --- | --- | --- | --- | --- | --- | --- |
|  | **Univariate** | | **Multivariate** | | **Univariate** | | | **Multivariate** | |
|  | **HR**  **(95% CI)** | ***P* valor** | **HR**  **(95% CI)** | ***P* valor** | **HR**  **(95% CI)** | | ***P* valor** | **HR**  **(95% CI)** | ***P* valor** |
| ≥65 years at diagnosis* | 2.1 (1.3-3.3) | 0.002 | 2.5 (1.4-4.3) | 0.001 | 3.4 (2.0-5.9) | | 0.000 | 3.9 (2.1-7.1) | 0.000 |
| PS ≥2 | 1.4 (0.8-2.4) | 0.186 | - | - | 1.6 (0.9-2.7) | | 0.117 | - | - |
| Anemia  (hemoglobin ≤10 g/dL) | 1.0 (0.6-1.6) | 0.994 | - | - | 0.9 (0.5-1.6) | | 0.749 | - | - |
| Hypercalcemia  (≥11 mg/dL) | 1.1 (0.6-2.1) | 0.790 | - | - | 1.2 (0.6-2.3) | | 0.670 | - | - |
| Kidney failure  (creatinine ≥2 mg/dL) | 1.7 (1.0-3.1) | 0.072 | - | - | 1.5 (0.8-2.8) | | 0.226 | - | - |
| Presence of lytic lesions | 1.0 (0.6-1.7) | 0.864 | - | - | 1.0 (0.6-1.8) | | 0.939 | - | - |
| Presence of plasmacytomas | 1.2 (0.6-2.4) | 0.657 | - | - | 0.7 (0.3-1.6) | | 0.396 | - | - |
| Higher levels of LDH | 1.7 (1.0-2.8) | 0.061 | - | - | 1.8 (1.0-3.2) | | 0.053 | - | - |
| β_2_ microglobulin ≥5 mg/dL | 1.7 (1.1-2.8) | 0.041 | 2.5 (1.4-4.4) | 0.002 | 1.5 (0.9-2.7) | | 0.137 | - | - |
| Bone marrow infiltration ≥60% | 0.9 (0.5-1.5) | 0.711 | - | - | 1.5 (0.8-2.7) | | 0.212 | - | - |
| Olygosecretory disease (paraprotein ≤1g/dL) | 1.0 (0.5-1.8) | 0.895 | - | - | 1.2 (0.6-2.2) | | 0.740 | - | - |
| Novel agents in first line | 0.8 (0.5-1.3) | 0.450 | - | - | 0.7 (0.4-1.2) | | 0.175 | - | - |
| To not undergo ASCT* | 2.5 (1.6-4.0) | 0.000 | - | - | 3.3 (2.0-5.7) | | 0.000 | - | - |
| To not achieve ≥PR after induction* | 4.2 (2.3-7.7) | 0.000 | 4.3 (2.2-8.4) | 0.000 | 3.7 (2.0-6.9) | | 0.000 | 4.8 (2.5-9.3) | 0.000 |
| To not achieve ≥CR after induction* | 2.0 (1.1-3.9) | 0.031 | - | - | 1.8 (0.8-4.0) | | 0.136 | - | - |

**Supplementary table 1.** Univariate and multivariate analysis in the t(11;14) group

PS: Performance status; LDH: lactate dehydrogenase; ASCT: autologous stem cell transplantation; PR: partial response; CR: complete response; HR: Hazard ratio; CI: confidence interval.

*To not undergoing ASCT and to not achieve ≥CR were not included in the multivariate analysis because of multicollinearity.
